# Adoption of immunotherapy in the community for patients diagnosed with metastatic melanoma

**DOI:** 10.1186/s40425-019-0782-y

**Published:** 2019-11-07

**Authors:** Marieke J. Krimphove, Karl H. Tully, David F. Friedlander, Maya Marchese, Praful Ravi, Stuart R. Lipsitz, Kerry L. Kilbridge, Adam S. Kibel, Luis A. Kluth, Patrick A. Ott, Toni K. Choueiri, Quoc-Dien Trinh

**Affiliations:** 1 Division of Urological Surgery and Center for Surgery and Public Health, Brigham and Women’s Hospital, Harvard Medical School, Boston, MA USA; 2 Department of Urology, University Hospital Frankfurt, Frankfurt am Main, Germany; 3 Department of Urology, Marien Hospital Herne, Ruhr-University Bochum, Herne, Germany; 4 Department of General Internal Medicine and Center for Surgery and Public Health, Brigham and Women’s Hospital, Harvard Medical School, Boston, MA USA; 5 Department of Medical Oncology, Dana Farber Cancer Institute, Harvard Medical School, Boston, MA USA

**Keywords:** Metastatic melanoma, Immunotherapy, Checkpoint inhibitors, Health services research, Ipilimumab

## Abstract

**Background:**

The introduction of immune checkpoint inhibitors has led to a survival benefit in patients with advanced melanoma; however data on the adoption of immunotherapy in the community are scarce.

**Methods:**

Using the National Cancer Database, we identified 4725 patients aged ≥20 diagnosed with metastatic melanoma in the United States between 2011 and 2015. Multinomial regression was used to identify factors associated with the receipt of treatment at a low vs. high immunotherapy prescribing hospital, defined as the bottom and top quintile of hospitals according to their proportion of treating metastatic melanoma patients with immunotherapy.

**Results:**

We identified 246 unique hospitals treating patients with metastatic melanoma. Between 2011 and 2015, the proportion of hospitals treating at least 20% of melanoma patients with immunotherapy within 90 days of diagnosis increased from 14.5 to 37.7%. The mean proportion of patients receiving immunotherapy was 7.8% (95% Confidence Interval [CI] 7.47–8.08) and 50.9% (95%-CI 47.6–54.3) in low and high prescribing hospitals, respectively. Predictors of receiving care in a low prescribing hospital included underinsurance (no insurance: relative risk ratio [RRR] 2.44, 95%-CI 1.28–4.67, *p* = 0.007; Medicaid: RRR 2.10, 95%-CI 1.12–3.92, *p* = 0.020), care in urban areas (RRR 2.58, 95%-CI 1.34–4.96, *p* = 0.005) and care at non-academic facilities (RRR 5.18, 95%CI 1.69–15.88, *p* = 0.004).

**Conclusion:**

While the use of immunotherapy for metastatic melanoma has increased over time, adoption varies widely across hospitals. Underinsured patients were more likely to receive treatment at low immunotherapy prescribing hospitals. The variation suggests inequity in access to these potentially life-saving drugs.

## Introduction

The incidence of melanoma is rising, with the majority of cases diagnosed at localized stages, with relatively high cure rates [1]. However, recurrent and metastatic melanoma is associated with a worse prognosis. The emergence of immune checkpoint inhibitors have ushered in a new era of therapy for recurrent and advanced melanoma and many other [2–4]. In early 2011, the Food and Drug Administration (FDA) approved ipilimumab, an antibody that blocks the inhibitory receptor CTLA-4 expressed on T cells, (the first immunotherapeutic drug in the class of immune checkpoint inhibitors) for the treatment of advanced stage melanoma [5]. Antibodies directed against another inhibitory receptor, programmed death 1 (PD-1) and PD-1 ligand either used as monotherapy or in combination with ipilimumab have demonstrated overall survival benefit compared to ipilimumab alone and chemotherapy and are now approved by regulatory agencies and standard of care for the treatment of a number of solid and hematologic malignancies including melanoma [3, 4].

While retrospective studies have confirmed the survival benefit with immune checkpoint inhibitors in the treatment of metastatic melanoma observed in prospective studies, [6] there are scarce data on the adoption of immunotherapy in the community. We therefore aimed to investigate the use of immunotherapy for metastatic melanoma across hospitals over time, and sought to identify factors associated with the receipt of immunotherapy in the community. We hypothesized that certain hospitals are better equipped than others to adopt these new therapies.

## Material and methods

### Data source

We queried the National Cancer Database (NCDB) to obtain data from patients seen at one of 1500 Commission on Cancer (CoC) accredited hospitals. The registry was established by the American Cancer Society and captures approximately half of melanoma cancer cases in the United States [7]. It contains sociodemographic and clinical data, including cancer characteristics and treatment information collected from trained data abstractors following standardized methodology.

### Study population

**Fig. 1:**
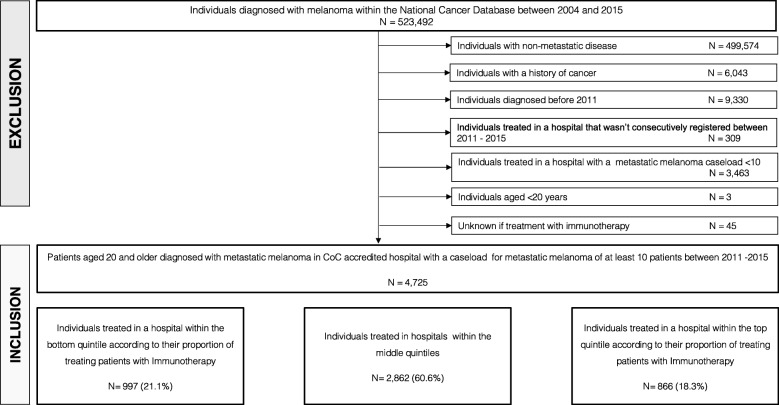
Flow-chart data selection

Individuals diagnosed with metastatic melanoma between 2011 and 2015 were identified according to World Health Organization ICD-O3 morphological codes for malignant melanoma as well as skin topographical codes (i.e., C44.0–44.9) as previously described [6]. Metastatic stage was defined according to the collaborative stage data collection system variables indicating metastatic disease and site at diagnosis as well as clinical or pathological metastatic stage according to the American Joint Committee on Cancer, 7th Edition. If information on lactate dehydrogenase (LDH) level was available and LDH was elevated, patients were categorized as metastatic stage IVM1c. In patients with no information on LDH level metastatic stage was categorized based on metastatic site only. Patients with conflicting information about metastatic status were excluded. We only included patients who were treated at CoC accredited facilities that were registered throughout the study period between 2011 and 2015. Furthermore, we excluded patients with a history of a non-melanoma cancer, and patients with missing information on immunotherapy. For confidentiality reasons, we excluded patients < 20 years of age and who were treated at facilities that treated less than 10 patients for metastatic melanoma between 2011 and 2015 (Fig. 1). In the NCDB, immunotherapy is recorded in a single treatment variable, however, since PD-1 inhibitors for advanced melanoma were approved by the FDA in late 2014, we assume that cases reporting the receipt of immunotherapy in those years were most likely ipilimumab monotherapy.

### Variables of interest - covariates

Patient level information included gender, age at diagnosis, race (white, black, other, unknown), year of diagnosis, health related and cancer related characteristics comprised by the Charlson Deyo Index (CCI; categorized into 0, 1, 2, ≥3), primary site of the tumor (head and neck, trunk, extremities, overlapping/unknown), histology (melanoma/not otherwise specified [NOS], nodular, lentigo, superficial, other/unknown), M stage including metastatic site (pM1/NOS, pM1a-c, brain involvement), Breslow depth and ulceration status (present, absent, unknown). Sociodemographic information contained primary insurance carrier (private, Medicaid, Medicare, other government payer [TRICARE, Military, VA and Indian/Public Health Service], uninsured, unknown), percentage of adults within patient’s ZIP code without a high school diploma (< 7%, 7–12.9%, 13–20.9%, ≥21%), ZIP code level median household income per year (<$38,000, $38,000–$47,999, $48,000–$62,999, or ≥ $63,000), and distance to the CoC facility. Facility level data included county type defined as an area-based measure of rurality and urban influence, using the typology published by the USDA Economic Research Service [8] (metropolitan, urban, rural, or unknown), census geographical region, and facility type categorized as Academic Program, Community Cancer Program, Comprehensive Community Cancer Program, Integrated Network Cancer Program, or other/unknown.

### Main outcome measure

The main outcome measure was the rate of use of immunotherapy in hospitals treating patients with metastatic melanoma. Therefore, all hospitals were ranked according to their proportion of patients treated with immunotherapy relative to their total metastatic melanoma caseload between 2011 and 2015. Similar to an established method of stratifying volumes, [9, 10] we divided hospitals into quintiles. The primary comparison of interest was between hospitals in the bottom and top quintiles, defined as low and high prescribing hospitals, respectively.

### Statistical analysis

First, in order to explore and describe the use of immunotherapy across hospitals over time, where time is defined as time since diagnosis, we looked at the proportion of hospitals treating at least 20% of patients with immunotherapy within 15 to 90 days from diagnosis in different diagnosis years, similar to work done by Keating et al.^19^. We based our threshold on the mean proportion of patients being treated with immunotherapy per hospital and year (20.6%) which thus represents the routine use across hospitals. To account for variation in facility caseloads over time, we determined the annual caseloads of metastatic melanoma, defined as the total volume of patients with metastatic melanoma treated at the treating facility in the year of the patient’s diagnosis [11, 12].

Second, baseline characteristics of patients treated at low vs. high prescribing hospitals were reported using medians and interquartile ranges (IQR) for continuous variables; categorical variables were presented using frequencies and proportions. Mann-Whitney U test and Pearson’s χ2 test were used to compare differences in continuous and categorical variables, respectively. Patients, who were treated in hospitals of the middle quintiles were excluded from baseline analyses.

Finally, to assess possible factors associated with the receipt of treatment in a low vs. high immunotherapy prescribing hospital, we fit a multinomial logistic regression, accounting for patients who were treated in hospitals of the middle quintiles, and setting high prescribing hospital as our reference group. To account for unmeasured differences between hospitals, all regression analyses were adjusted for facility-level clustering [13].

All statistical analyses were performed using Stata v.13.0 (StataCorp, College Station, TX, USA). Two-sided statistical significance was defined as *p* < 0.05. Before conducting the study, we obtained a review board waiver from our institution.

## Results

### Use of immunotherapy over time

**Fig. 2:**
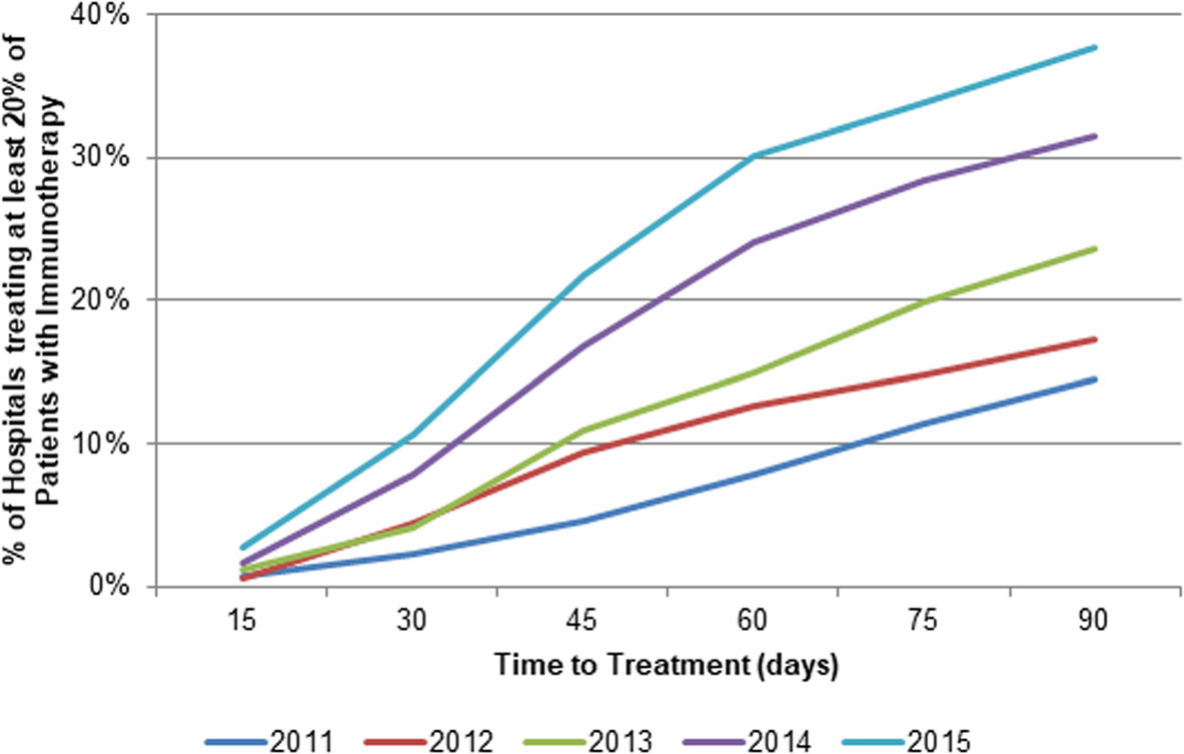
Proportion of hospitals treating at least 20% of Patients with Immunotherapy within 15 to 90 days stratified by year of diagnosis (2011–2015)

Figure 2 depicts the use of immunotherapy across hospitals over time, stratified by diagnosis year. Of all hospitals that cared for patients with metastatic melanoma diagnosed in 2011, 0.7% used immunotherapy in at least 20% of all patients within 15 days from diagnosis increasing to 14.5% within 90 days from diagnosis. The slope was significantly steeper in later years, with the proportion of hospitals treating at least 20% of patients within 15 and 90 days increasing from 2.8 to 37.7%, respectively, in 2015.

### Variation in the use of immunotherapy across hospitals

**Fig. 3:**
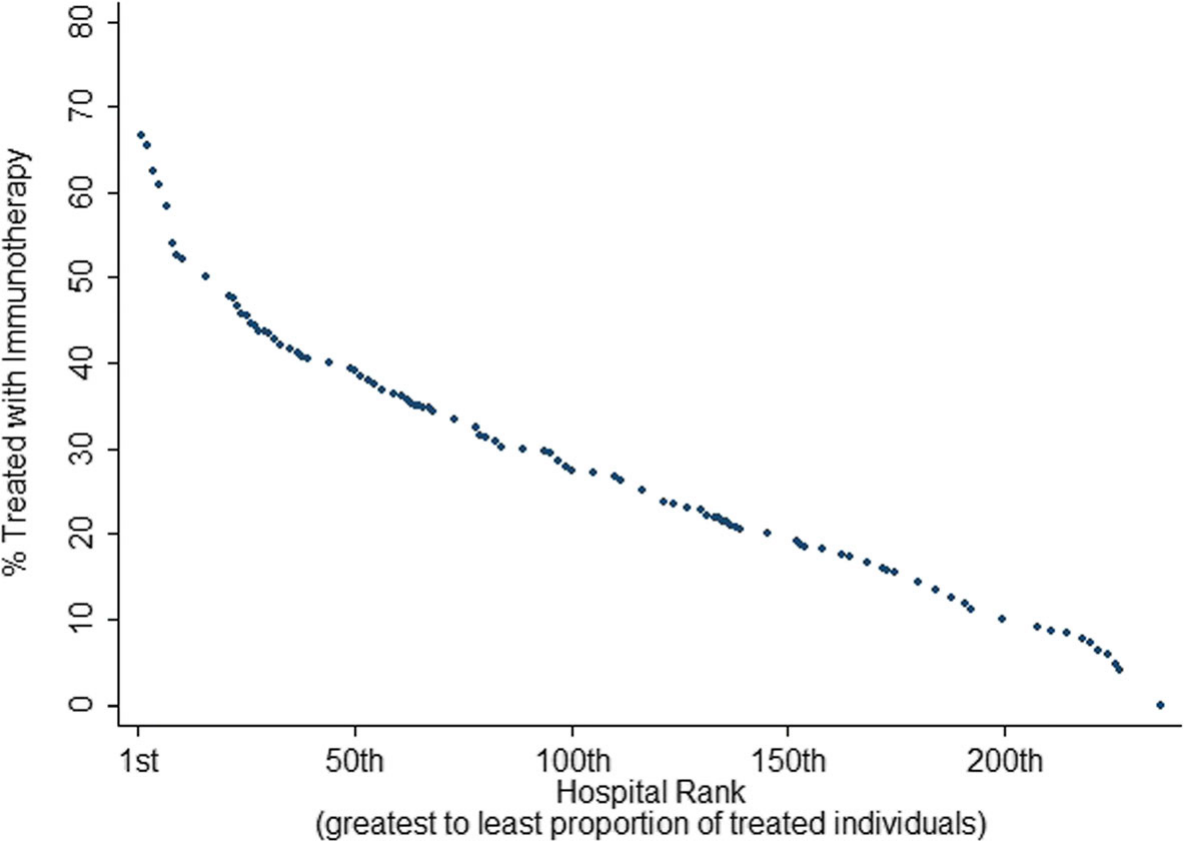
Facilities (*n* = 246) ranked according to their proportion of treating patients diagnosed with metastatic melanoma with immunotherapy between 2011 and 2015

We identified 246 unique hospitals treating at least 10 patients diagnosed with metastatic melanoma between 2011 and 2015. The overall proportion of patients treated with immunotherapy was 23.8%, ranging from 0 to 75% across hospitals. The mean proportion of patients receiving immunotherapy was 7.8% (95% Confidence Interval [CI] 7.47–8.08) and 50.9% (95% CI 47.6–54.3) at low and high prescribing hospitals, respectively (Fig. 3).

### Baseline characteristics of individuals treated at low vs. high prescribing hospitals

A total of 4725 patients met inclusion criteria, 997 (21.1%) of which were treated in low prescribing hospitals, and 866 (18.3%) in high prescribing hospitals. Baseline characteristics of patients treated at low vs. high prescribing hospitals are summarized in Table 1. Patients treated at low prescribing hospitals were older (81–90 years: 16.8% vs. 8.6%, *p* < 0.001), sicker (CCI of 1: 18.4% vs. 12.7%, p < 0.001), poorer (Median county-level income ≥$63,000: 32% vs. 45.6%, *p* = 0.021), less educated (residence in an area where < 7% have no high school diploma: 22.4% vs. 36.8%, p < 0.001), and more often had no insurance (7.5% vs. 3.0%, p < 0.001). Low prescribing hospitals less often were academic centers (34.4% vs. 82.6%, p < 0.001).

**Table 1:** Baseline Characteristics of Patients with metastatic melanoma treated in low vs. high immunotherapy prescribing hospitals between 2011 and 2015

	Low prescribing Hospital *N* = 997 (53.5%)	High prescribing Hospital *N* = 866 (46.5%)	*p*-value
Age, n (%)			< 0.001
≤ 30	19 (1.9)	21 (2.4)	
31–40	41 (4.1)	66 (7.6)	
41–50	98 (9.8)	105 (12.1)	
51–60	181 (18.2)	211 (24.4)	
61–70	286 (28.7)	240 (27.7)	
71–80	205 (20.6)	149 (17.2)	
81–90	167 (16.8)	74 (8.6)	
Gender, n (%)			0.430
Female	300 (30.1)	277 (32.0)	
Male	697 (69.9)	589 (68.0)	
Race, n (%)			0.743
White	963 (96.6)	832 (96.1)	
Black	13 (1.3)	15 (1.7)	
Other	21 (2.1)	19 (2.2)	
Year of Diagnosis, n (%)			0.481
2011	188 (18.9)	176 (20.3)	
2012	186 (18.7)	164 (18.9)	
2013	186 (18.7)	182 (21.0)	
2014	213 (21.4)	175 (20.2)	
2015	224 (22.5)	169 (19.5)	
Charlson Deyo Index, n (%)			*< 0.001*
0	740 (74.2)	723 (83.5)	
1	183 (18.4)	110 (12.7)	
2	50 (5.0)	24 (2.7)	
≥ 3	24 (2.4)	9 (1.0)	
Primary Site, n (%)			0.337
Head and Neck	81 (8.1)	96 (11.1)	
Trunk	146 (14.6)	119 (13.7)	
Extremities	120 (12.0)	108 (12.5)	
overlapping	650 (65.2)	543 (62.7)	
Histology, n (%)			*0.035*
Melanoma, NOS	874 (87.6)	701 (81.0)	
Nodular	49(4.9)	76 (8.8)	
Lentigo	5 (0.5)	14 (1.6)	
Superficial spreading	25 (2.5)	33 (3.8)	
Acral lentiginous	2 (0.2)	9 (1.0)	
other	42 (4.2)	33 (3.8)	
Metastatic stage, n (%)			*0.020*
M1, NOS	89 (8.9)	41 (4.7)	
M1a	136 (13.6)	110 (12.7)	
M1b, lung	160 (16.1)	105 (12.1)	
M1c, visceral	463 (46.4)	530 (61.2)	
Brain involvement	149 (14.9)	80 (9.2)	
Ulceration, n (%)			0.333
No ulceration	199 (20.0)	193 (22.3)	
Ulceration present	135 (13.5)	135 (15.6)	
unknown	663 (66.5)	538 (62.1)	
Breslow depth (continuous)			0.559
Insurance, n (%)			*< 0.001*
Private	318 (31.9)	415 (47.9)	
Medicare	476 (47.7)	315 (36.4)	
Medicaid	102 (0.2)	55 (6.4)	
Other Government	18 (1.8)	10 (1.2)	
No insurance	75 (7.5)	26 (3.0)	
unknown	8 (0.8)	45 (5.2)	
Income*, n (%)			*0.021*
≥ $ 63,000+	319 (32.0)	395 (45.6)	
$ 48,000 – 62,999	278 (27.9)	238 (27.5)	
$ 38,000 – 47,999	248 (24.9)	164 (18.9)	
< $ 37,000	147 (14.7)	68 (7.9)	
unknown	5 (0.5)	1 (0.1)	
Education*^,^**, n (%)			*< 0.001*
≥ 21%	174 (17.5)	84 (9.7)	
13–20.9%	250 (25.1)	172 (19.9)	
7–12.9%	347 (34.8)	290 (33.5)	
< 7%	223 (22.4)	319 (36.8)	
unknown	3 (0.3)	1 (0.1)	
Great Circle Distance, n (%)			*< 0.001*
< 12.5mi	531 (53.3)	268 (31.0)	
12.5–49.9mi	352 (35.1)	347 (40.1)	
≥ 50mi	112 (11.2)	250 (28.9)	
unknown	2 (0.2)	1 (0.1)	
Facility Location, n (%)			*0.017*
Northeast	121 (12.1)	289 (33.4)	
South	509 (51.1)	171 (19.8)	
Midwest	90 (9.0)	129 (14.9)	
West	222 (22.3)	199 (23.0)	
unknown	55 (5.5)	78 (9.0)	
Facility Type, n (%)			*< 0.001*
Academic	324 (34.4)	651 (82.6)	
CCCP	472 (50.1)	113 (14.3)	
INCP	146 (15.5)	24 (3.1)	
County, n (%)			0.637
Metro	838 (84.1)	753 (87.0)	
Urban	116 (11.6)	89 (9.9)	
Rural	18 (1.8)	8 (0.9)	
unknown	25 (2.5)	19 (2.2)	

Abbreviations: NOS = not otherwise specified, mi = miles; CCCP = comprehensive community cancer program; INCP = integrated network cancer program

Significant *p*-values in *italic*

*ZIP-code level variable

**Percentage of residents in home county with no high school degree from 2012 American County Survey Data

**Table 2:** Multinomial logistic regression predicting treatment in a low vs. high immunotherapy prescribing hospital (accounting for the middle quintiles)

	Relative risk ratio	95% Confidence Interval	p-value
Age			
≤ 30	Ref.		
41–50	1.04	0.20–5.32	0.964
51–60	0.84	0.17–4.18	0.834
61–70	1.28	0.25–6.49	0.766
71–80	1.19	0.25–5.68	0.831
81–90	2.24	0.46–10.9	0.318
Gender			
male	Ref.		
female	0.86	0.62–1.18	0.346
Race			
White	Ref.		
Black	0.36	0.11–1.14	0.081
Other/unknown	1.97	0.71–5.46	0.193
Year of Diagnosis			
2011	Ref.		
2012	1.28	0.82–2.01	0.287
2013	1.08	0.69–1.70	0.728
2014	1.40	0.89–2.22	0.147
2015	1.83	1.17–2.87	*0.008*
Charlson Deyo Index			
0	Ref.		
1	1.11	0.73–1.71	0.623
2	1.30	0.50–2.21	0.888
> =3	1.93	0.77–4.87	0.162
Primary Site			
Head and Neck	Ref.		
trunk	1.41	0.74–2.71	0.294
extremities	1.30	0.72–2.33	0.379
overlapping	1.25	0.60–2.63	0.550
Histology			
Melanoma, NOS	Ref.		
nodular	0.78	0.44–1.39	0.396
Lentigo	0.35	0.04–3.44	0.369
superficial	0.87	0.38–2.00	0.749
Acral	0.47	0.07–3.01	0.424
other	0.91	0.43–1.93	0.798
Metastatic stage			
M1, NOS	Ref.		
M1a	0.42	0.15–1.17	0.097
M1b, lung	0.39	0.14–1.10	0.075
M1c, visceral	0.22	0.08–0.62	*0.004*
Brain involvement	0.44	0.15–1.23	0.116
Ulceration			
No ulceration	Ref.		
ulceration	0.88	0.54–1.46	0.631
unknown	0.63	0.35–1.15	0.132
**Breslow** (continuous)	1.00	1.00–1.00	0.141
Insurance			
Private	Ref.		
Medicare	1.13	0.74–1.71	0.576
Medicaid	2.10	1.12–3.92	*0.020*
Other	1.11	0.34–3.63	0.850
No insurance	2.44	1.28–4.67	*0.007*
unknown	0.28	0.07–1.16	0.080
Income*			
≥ $63,000	Ref.		
$48,999–$62,999	1.25	0.65–2.40	0.509
$38,000–$47,999	0.93	0.36–2.41	0.887
< $38,000	1.71	0.57–5.14	0.339
unknown	0.29	0.02–3.54	0.333
Education*^,^**			
≥ 21%	Ref.		
13%-20,9%	1.14	0.55–2.37	0.730
7–12,9%	0.97	0.40–2.33	0.943
< 7%	0.61	0.21–1.76	0.360
unknown	6.10	0.38–98.55	0.203
Distance			
< 12.5mi	Ref.		
12.5-50mi	0.58	0.38–0.88	*0.011*
≥ 50mi	0.14	0.07–0.30	*< 0.001*
unknown	0.38	0.07–2.23	0.285
Facility Location			
Northeast	Ref.		
South	5.06	0.98–26.03	0.052
Midwest	0.97	0.15–6.06	0.973
West	1.81	0.35–9.30	0.475
Facilitytype			
Academic	Ref.		
CCCP	5.18	1.69–15.88	*0.004*
INCP	6.60	1.06–41.14	*0.043*
County			
Metro	Ref.		
Urban	2.58	1.34–4.96	*0.005*
Rural	1.93	0.32–11.74	0.476
unknown	1.25	0.45–3.45	0.671

Abbreviations: NOS = not otherwise specified, mi = miles; CCCP = comprehensive community cancer program; INCP = integrated network cancer program

Significant *p*-values in *italic*

*ZIP-code level variable

**Percentage of residents in home county with no high school degree from 2012 American County Survey Data

### Factors associated with receipt of treatment at low vs. high immunotherapy prescribing hospitals

Table 2 shows predictors of receiving care in a low prescribing hospital including Medicaid insurance (relative risk ratio [RRR] 2.10, 95% CI 1.12–3.92, *p* = 0.020) or no insurance (RRR 2.44, 95% CI 1.28–4.67, *p* = 0.007) relative to private insurance, and absence of visceral metastases (RRR 0.22, 95% CI 0.08–0.62, *p* = 0.004). Also, patients with a long travel distance were less likely to be treated at low prescribing hospitals (≥50mi: RRR 0.14, 95% CI 0.07–0.3, p < 0.001). On a facility level, low prescribing hospitals were more likely to be a Comprehensive Community Cancer Program (RRR 5.18, 95%CI 1.69–15.88, p = 0.004) relative to academic facilities and more likely to be located in urban areas (RRR 2.58, 95% CI 1.34–4.96, *p* = 0.005) relative to metropolitan areas.

## Discussion

We herein demonstrate not only how the use of immunotherapy for metastatic melanoma has spread over time but also how its implementation has varied across hospitals and what factors predict treatment at hospitals with low vs. high use of immunotherapy. Since the approval of ipilimumab as the first immunotherapeutic drug of its kind in 2011, immunotherapy has rapidly evolved and now represents first or second-line therapy for a variety of cancers [14, 15]. However, as demonstrated by our finding of significant facility-level variation in immunotherapy uptake, it is conceivable that the enormous economic burden of this new therapy [16] is hampering comprehensive implementation across hospitals.

When considering the general use of immunotherapy from the time of its first approval in 2011 to recent years, we found a gradual uptake in the use of immunotherapy across hospitals (Fig. 3) that is consistent with adoption curves witnessed with other novel drugs or devices [17]. The proportion of hospitals treating at least 20% of their patients with immunotherapy for metastatic melanoma within 90 days of diagnosis was approximately 2.5 times higher in 2015 compared to 2011. This trend is likely to continue as familiarity with targeted therapies increases among healthcare professionals [18].

Despite level-one evidence demonstrating a survival benefit associated with the use of immunotherapy in the treatment of metastatic melanoma, we noted significant facility-level variation in immunotherapy uptake [5]. Facility-level rates of immunotherapy use in high-prescribing hospitals approached 50%, compared to just 8% among low prescribing hospitals. Our results corroborate results from investigations regarding variations in the use of new therapeutics in other cancers [19]. Collectively, these results suggest that non-clinical predictors of care such as facility type may be contributing to care inequity that disproportionately affects underserved communities. Non-adherence to clinical guidelines and recommendations is a phenomenon that has repeatedly been shown across a variety of specialties and conditions (including melanoma), [20, 21] which in turn may affect clinical prognosis [22, 23]. Consequently, it is critical that providers and policymakers alike identify and eliminate drivers of healthcare that is either not indicated or inadequate.

Patient and physician-level factors must also be considered as a source of the variation observed in our study [20]. A lack of experience and poor access to information regarding the appropriate use of immunotherapy may discourage physician uptake, particularly given that immune-related toxicities can result in mortality and their management often requires specific expertise [24]. From the patient’s perspective, compliance with these novel drugs, especially in the context of adverse effects, requires adequate financial stability, as well as family/social support. Similarly, low prescribing hospitals were more likely to be non-academic centers that may not have early access to immunotherapy in the context of clinical trials which precede FDA approval and wider access to new agents. More than 80% of hospitals treating the highest proportion of patients with immunotherapy were academic. These academic institutions have greater access to clinical trials that may provide immunotherapy before FDA approval. Access to drugs in a clinical trial setting is likely to facilitate rapid implementation and routine use of new drugs after FDA approval because physicians will have greater familiarity with managing immune-related toxicities.

Financial aspects potentially affecting the care setting for metastatic melanoma patients must also be considered as evident by our finding that underinsured patients with Medicaid insurance or no insurance had a much higher probability of being treated in a low prescribing hospital. While drug coverage (as provided by Medicaid) is one aspect of the question, there are other factors around the treatment of the patient including payments to providers and hospitals that will be impacted by patient insurance. While most providers and hospitals – at least deliberately – do not select patients according to their insurance for the simple goal of maximizing profit, there is certainly a larger scale systemic incentive to do so. Our findings are consistent with prior work showing that underserved populations experience lower quality care across a variety of health care settings [25, 26]. The cost-intensive nature of immunotherapy is likely to exacerbate already observed health inequities experienced by the socioeconomically disadvantaged as hospitals and patients with lower means to pay for adequate treatment and lack of resources may affect treatment uptake and adherence [27]. Indeed, the administration of novel immunotherapy requires supplemental resources; in addition to the costs for the drug itself, there are added expenditures related to implementing support and pharmacy teams are required to correctly treat patients that are more easily borne by large academic centers.

Interestingly, the only clinical factor associated with lower odds of being treated at a low-prescribing hospital was the presence of visceral metastatic disease. However, factors classically used to define patient eligibility to systematic treatment, such as age or comorbidities, [28] were not different between the hospitals. There is evidence that better outcomes can be achieved when patients with complex diseases receive care at more specialized hospitals, supporting the concept of centralization [29]. It is possible that care for patients with more advanced disease may be more likely to be transferred to more experienced hospitals, there is no other clinical factor explaining differences in the use of immunotherapy.

We acknowledge that our work has some limitations. First, we are unable to adjust for intrinsic confounding given the retrospective observational nature of our study. Second, the database we used, NCDB, is a hospital-based registry that contains only information on patients treated at CoC-accredited hospitals. Our results may therefore not be representative for patients being treated outside of these facilities. Third, the NCDB does not capture the type or dosage of immunotherapy administered and approvals of PD-1/PD-L1 inhibitors fall in the latter time frame of our investigation. As a result, our data are more likely to reflect adoption of ipilimumab than adoption of nivolumab and pembrolizumab though we cannot distinguish use of individual immunotherapy agents. For the same reason, it is possible that some patients received experimental immunotherapy agents on clinical trials that were not FDA approved at the time of their administration. Although it is beyond the scope of our current investigation, it will be crucial to expand our next analysis to the timeframe between 2015 and 2018 to explore the broadening indications for immunotherapy. Greater familiarity with these agents with time may lead to more rapid adoption of immunotherapy in the community and increased use in non-academic centers.

## Conclusion

While the use of immunotherapy for metastatic melanoma has increased over time, adoption varies widely across hospitals. Underinsured patients were more likely to receive treatment at low immunotherapy prescribing hospitals. The variation suggests inequity in access to these potentially life-saving drugs.

## Data Availability

The data that support the findings of this study are available from the American College of Surgeons but restrictions apply to the availability of these data, which were used under license for the current study, and so are not publicly available.

## Abbreviations

CCI: Charlson-Deyo-Index
CI: Confidence Interval
CoC: Comission on Cancer
CCCP: Comprehensive Community Cancer Program
FDA: Food and Drug Administration
INCP: Integrated Network Cancer Program
IQR: Interquartile Range
LDH: Lactate dehydrogenase
NCDB: National Cancer Database
NOS: Not otherwise specified
RRR: Relative risk ratio
